# Spleen Tyrosine Kinase Regulates AP-1 Dependent Transcriptional Response to Minimally Oxidized LDL

**DOI:** 10.1371/journal.pone.0032378

**Published:** 2012-02-22

**Authors:** Soo-Ho Choi, Philipp Wiesner, Felicidad Almazan, Jungsu Kim, Yury I. Miller

**Affiliations:** Department of Medicine, University of California San Diego, La Jolla, California, United States of America; University of California Merced, United States of America

## Abstract

Oxidative modification of low-density lipoprotein (LDL) turns it into an endogenous ligand recognized by pattern-recognition receptors. We have demonstrated that minimally oxidized LDL (mmLDL) binds to CD14 and mediates TLR4/MD-2-dependent responses in macrophages, many of which are MyD88-independent. We have also demonstrated that the mmLDL activation leads to recruitment of spleen tyrosine kinase (Syk) to TLR4 and TLR4 and Syk phosphorylation. In this study, we produced a macrophage-specific Syk knockout mouse and used primary Syk^−/−^ macrophages in our studies. We demonstrated that Syk mediated phosphorylation of ERK1/2 and JNK, which in turn phosphorylated c-Fos and c-Jun, respectively, as assessed by an *in vitro* kinase assay. c-Jun phosphorylation was also mediated by IKKε. c-Jun and c-Fos bound to consensus DNA sites and thereby completed an AP-1 transcriptional complex and induced expression of CXCL2 and IL-6. These results suggest that Syk plays a key role in TLR4-mediated macrophage responses to host-generated ligands, like mmLDL, with subsequent activation of an AP-1 transcription program.

## Introduction

Spleen tyrosine kinase (Syk) is best known as a tyrosine kinase involved in signaling initiated by B cell receptor, T cell receptor or Fc receptor. Ligand binding to these receptors leads to recruitment of signaling proteins with immunoreceptor tyrosine-based activation motifs (ITAMs). Ensuing phosphorylation of tandem tyrosines in an ITAM leads to the ITAM binding with Syk via Syk's tandem SH2 domains, with subsequent Syk activation. Activated Syk directly binds to Vav, PLCγ, PI3K, and SLP76/SLP65 and engages a plethora of other signaling intermediates. Resulting cellular responses range from cytoskeletal changes and ROS production to cell differentiation, proliferation and survival [1]. In addition to the SH-2-mediated binding to ITAMs, Syk also binds to β-integrins via a site distinct from the site of phospho-tyrosine binding [2], [3] and thus, coordinates integrin- and ITAM-dependent signaling cascades.

We have recently demonstrated, using yeast-two-hybrid and immunoprecipitation assays, that Syk constitutively binds to the intracellular domain of toll-like receptor-4 (TLR4) [4], [5]. This binding is further increased and both TLR4 and Syk become phosphorylated in macrophages stimulated with a host-derived TLR4 ligand, minimally oxidized low-density lipoprotein (mmLDL). Further downstream from Syk, mmLDL activates the Vav-Ras-Raf-MEK1-ERK1/2 pathway, small GTPases Cdc42, Ras and Rho, and phosphorylates paxillin, which collectively lead to actin polymerization and extensive membrane ruffling, culminating in robust macropinocytosis. This mmLDL-induced and TLR4/Syk-dependent macropinocytosis is suggested to constitute an important mechanism of excessive lipid accumulation in macrophages, resulting in formation of lipid-laden macrophage foam cells, a hallmark of atherosclerosis [4]–[8].

Interestingly, unlike LPS, the mmLDL activation of TLR4 does not engage MyD88-dependent pathways [4], [5]. This makes the Syk-dependent signaling a central component of mmLDL-induced macrophage activation via TLR4. In the present work, we generated Syk^flox/flox^/LysM-Cre mice and performed the majority of experiments with primary macrophages derived from bone marrow in which Syk expression was reduced by 60–70%, to study the role of Syk in transcriptional regulation. We demonstrate that Syk regulates ERK1/2-dependent phosphorylation of c-Fos and JNK/IKKε-dependent phosphorylation of c-Jun, as well as c-Fos and c-Jun binding to DNA and AP-1-dependent expression of CXCL2 and IL-6. These results suggest the role of Syk in TLR4-mediated chronic inflammation induced by modified host ligands, as it may relate to inflammation in atherosclerotic lesions induced by oxidized LDL.

## Materials and Methods

### Ethics Statement

All animal experiments were performed according to the NIH guidelines and were approved by the Animal Subjects Committee of the UC San Diego (protocol S04155). Human plasma, used for LDL isolation, was obtained from normal volunteers according to a protocol approved by the UC San Diego Human Research Protection Program (project #071402).

### Animals

Syk^flox/flox^ mice were kindly provided by Alexander Tarakhovsky from the Rockfeller University [9]. LysM-Cre mice were purchased from the Jackson Laboratory. Myeloid cell lineage specific Syk knockdown mice, thereafter referred to as Syk^−/−^, were generated by breeding Syk^flox/flox^ mice with LysM-Cre mice, the latter express myeloid cells specific lysozyme 2 promoter-driven Cre recombinase. Littermate Syk^flox/flox^/Cre(−) mice, thereafter referred to as wild type (WT), were used as a control in all experiments. In addition, we used MyD88^−/−^ mice on the C57BL6 background [10], kindly provided by Dr. Shizuo Akira from Research Institute for Microbial Diseases, Osaka University, and wild type C57BL6 mice as a control.

### Cell culture

The majority of experiments were performed with bone marrow derived macrophages (BMDM) obtained from bone marrow cells isolated from Syk^flox/flox^/Cre(+) (Syk^−/−^) and Syk^flox/flox^/Cre(−) (WT) mice and differentiated with macrophage colony stimulating factor (L929 conditioned medium) according to published protocols [11]. Briefly, after mice were sacrificed, tibias and femurs were dissected and washed with a BMDM growth media (10% FBS, 50 µg/ml gentamicin and supernatant from L929 cells) to suspend cells. The cells were placed in 10-cm Petri dishes and maintained in a humidified 37°C incubator for 5 days to allow differentiation. Resident peritoneal mouse macrophages were isolated by peritoneal lavage, without any prior elicitation. Isolated peritoneal macrophages were plated with DMEM containing 20% FBS and 50 µg/ml gentamicin. After 3 hours, floating cells were removed and attached cells were further incubated with 0.5% FBS/DMEM overnight.

Other cell types in which we achieved Syk loss- or gain-of-function were used to validate the results obtained with Syk^−/−^ BMDM. Chinese hamster ovary-K1 cells (CHO) were from American Type Culture Collection (ATCC) and maintained in Dulbecco's modified Eagle's medium (DMEM)/F12 supplemented with 10% fetal bovine serum (FBS) and 50 µg/ml gentamicin. Murine macrophage-like J774A.1 cell lines stably expressing Syk-specific or scrambled shRNA [5] were cultured in DMEM supplemented with 10% FBS, 50 µg/ml gentamicin and 0.8 mg/ml of G418 (Calbiochem) to maintain selection. A murine fibroblast cell line stably expressing human 15-lipoxygenase [12] was cultured in 10% FBS and 50 µg/ml gentamicin with 0.5 mg/ml of G418. Ba/F3 cells stably expressing GFP-TLR4 and Flag-TLR4 [13], [14] were cultured in RPMI1640 medium (Invitrogen) containing 70 units/ml recombinant murine interleukin-3, 10% heat-inactivated FBS, 100 units/ml penicillin, and 100 µg/ml streptomycin (Invitrogen).

### Antibodies

Two monoclonal antibodies to mouse TLR4/MD-2 antibodies, UT12 and UT18, were from eBioscience. An isotype mouse IgG3κ control was from BioLegend Antibodies specific to Syk, JNK, and IKKε were from Santa Cruz Biotechnology; ERK1/2, p-ERK1/2, p-JNK, p-c-Jun, p-p65, and GAPDH from Cell Signaling Technology; GFP from Abcam; and Flag from Sigma-Aldrich.

### LDL preparation and modification

LDL (density = 1.019–1.063 g/ml) was isolated from pooled human plasma by sequential ultracentrifugation [15]. Plasma was obtained from normal volunteers according to a protocol approved by the UCSD Human Research Protection Program (Project #071402). Native and modified LDL preparations were tested for possible endotoxin contamination using a Limulus Amoebocyte Lysate kit (Cambrex, Walkersville, MD). Only preparations with endotoxin <2.5 pg/ml at a final dilution were used in experiments. To produce mmLDL, we incubated 50 µg/ml of native LDL in serum-free DMEM for 18 hours with murine fibroblasts overexpressing human 15-lipoxygenase [16], [17]. To produce OxLDL, 100 µg/ml of native LDL was incubated with 10 µM CuSO_4_ for 18 hours at 37°C and then concentrated to 1 mg/ml [18]. The extent of LDL oxidation was assessed by measuring thiobarbituric acid reactive substances (typically, more than 30 nmol/mg protein in OxLDL).

### Recombinant adenovirus

Recombinant adenovirus expressing Cre recombinase was kindly provided by Byungkwan Lim (Kirk Knowlton's lab, UC San Diego). Recombinant adenoviruses were expanded in HEK293 cells (from ATCC). Isolated peritoneal macrophages were infected with recombinant adenovirus expressing Cre recombinase at 500 multiplicity of infection (MOI) for 48 hours. A recombinant adenovirus expressing GFP was used as control. After infection, cells were treated with media or mmLDL (50 µg/ml) and then cells were harvested.

### Real-time quantitative PCR (qPCR)

Total RNA was isolated from 1×10^6^ cells using RNeasy columns (Qiagen). Isolated RNA was reverse transcribed using a First Strand Synthesis kit (Invitrogen) following manufacturer's protocol. Probes, primers and reagents for TagMan qPCR were purchased from Applied Biosystems. qPCR analysis was performed using a Rotor Gene Q thermocycler (Qiagen).

### Cytokine ELISA

Cells (0.2×10^6^) were plated overnight and then incubated with media alone (DMEM supplemented with 5% lipoprotein-deficient serum (LPDS)) or in the presence of 50 µg/ml mmLDL for 6 hours or 24 hours. Supernatants were collected and centrifuged at 10,000 rpm for 5 min to remove floating cells. Levels of MIP-2 (CXCL2) and IL-6 secreted proteins were measured in ELISA using reagents from R&D Systems.

### Chemiluminescent assay for transcription factor/DNA binding

A chemiluminescent transcription factor/DNA binding assay was performed as described previously [19]. Briefly, cellular nuclear extracts were isolated using a Nuclear Extraction kit (Active Motif). The DNA binding of transcription factors (c-Jun, c-Fos, and p65) was measured using an Active Motif's TransAM kit (Active Motif).

### Luciferase reporter assay

CHO cells were transfected with 1 µg of Syk expression plasmid (kindly provided by Yun Soo Bae, Ewha Womans University, Korea), 0.2 µg of AP-1-luciferase plasmid (provided by Byungkwan Lim and Kirk Knowlton, UC San Diego), and 0.2 µg of β-galactosidase plasmid. Thirty six hours after transfection, cells were stimulated with 50 µg/ml mmLDL or media alone for 6 hours. Luciferase activity was normalized to β-galactosidase activity.

### Immunoblot analysis

Cells were lysed with an ice-cold lysis buffer (50 mM Tris-HCl, pH 7.5, 1% NP40, 150 mM NaCl, 1 mM EDTA, 1 mM EGTA, 5 mM Na_3_VO_4_, 1 mM NaF, and protease inhibitor cocktail from Sigma). Protein content was measured by a DC protein assay kit (BioRad) and equal protein amounts were loaded and run on a 4–12% PAGE gel (Invitrogen) and then transferred to a PVDF membrane (Invitrogen). The membranes were incubated with primary antibodies overnight at 4°C, followed by incubation with secondary antibodies conjugated with HRP for 1 hour at room temperature. The antibody-bound proteins were detected using a Super Signal West Dura substrate (Pierce) with an OptiChemHR Imaging System (UVP).

### *In vitro* kinase assay

J774 cells or BMDM were lysed in 600 µl of cell lysis buffer (20 mM Tris-HCl, pH 7.4, 150 mM NaCl, 1 mM EDTA, 1 mM EGTA, 1 mM β-glycerophosphate, 1 mM Na_3_VO_4_, 1% Triton X-100, 2.5 mM sodium pyrophosphate, protease inhibitor cocktail). Cell lysates were immunoprecipitated with either JNK, IKKε, or ERK1/2 antibodies overnight at 4°C, and then incubated further with protein A Sepharose for 1 h at 4°C. Immune complexes were washed twice with the lysis buffer and then washed twice with a kinase buffer (20 mM HEPES, pH 7.4, 1 mM MnCl_2_, 5 mM MgCl_2_, 10 mM β-glycerophosphate, 0.1 mM sodium orthovanadate, 2 mM NaF, 1 mM DTT). Samples were incubated with 5 µCi of [γ-^32^P]-ATP (NEN Life Science) and 2 µg of purified GST-c-Jun (1–79 aa) or GST-c-Fos (300–380 aa) in 25 µl of kinase buffer for 30 min at 30°C. (The GST-c-Jun and c-Fos cDNA were kindly provided by Michael Karin, UC San Diego). The reactions were stopped by adding LDS sample buffer (Invitrogen), the samples were run on a Nu-PAGE gel (Invitrogen), and phosphorylated proteins were detected by autoradiography.

### Oil red O staining

BMDM were stained for neutral lipid with Oil Red O and hematoxylin, as described previously [5]. Briefly, cells were incubated with media alone or 50 µg/ml mmLDL plus 200 µg/ml native LDL. After 40 hours, cells were washed, fixed and stained with Oil Red O and hematoxylin.

### Statistical analysis

Graphs represent means ± standard error from 2–4 independent experiments. Significance of differences was calculated using one-way ANOVA.

## Results

### Generation of myeloid cell-specific Syk knockout mice

**Figure 1:**
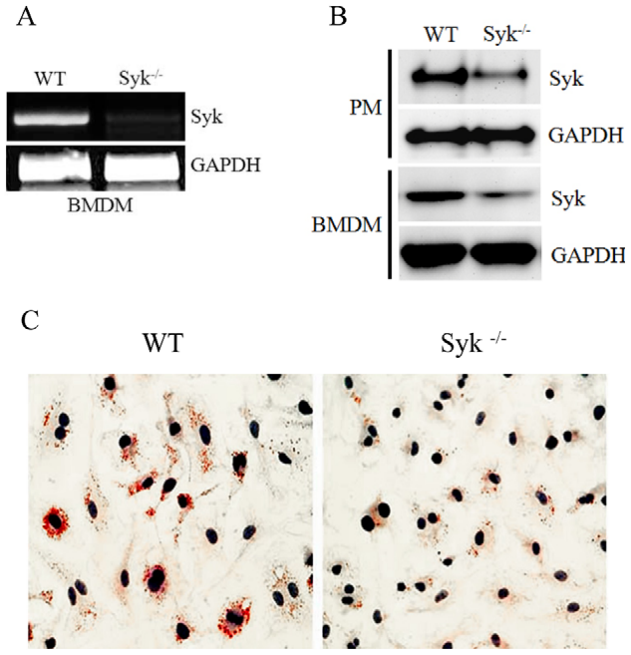
Reduced Syk expression in macrophages from Syk^flox/flox^/LysM-Cre mice. (**A**) Total RNA was isolated from BMDM of Syk^flox/flox^/LysM-Cre(−) (WT) and Syk^flox/flox^/LysM-Cre(+) (Syk^−/−^) mice, and RT-PCR with Syk and GAPDH primers was performed. (**B**) Lysates of resident peritoneal macrophages (PM) and BMDM were run on SDS-PAGE, transferred to a membrane and immunoblotted with anti-Syk and anti-GAPDH antibodies. (**C**) WT and Syk^−/−^ BMDM were incubated with 50 µg/ml mmLDL (to induce macropinocytosis) and 200 µg/ml of native LDL (lipid carrier) for 40 hours. The cells were stained for neutral lipid with Oil Red O.

**Figure 2:**
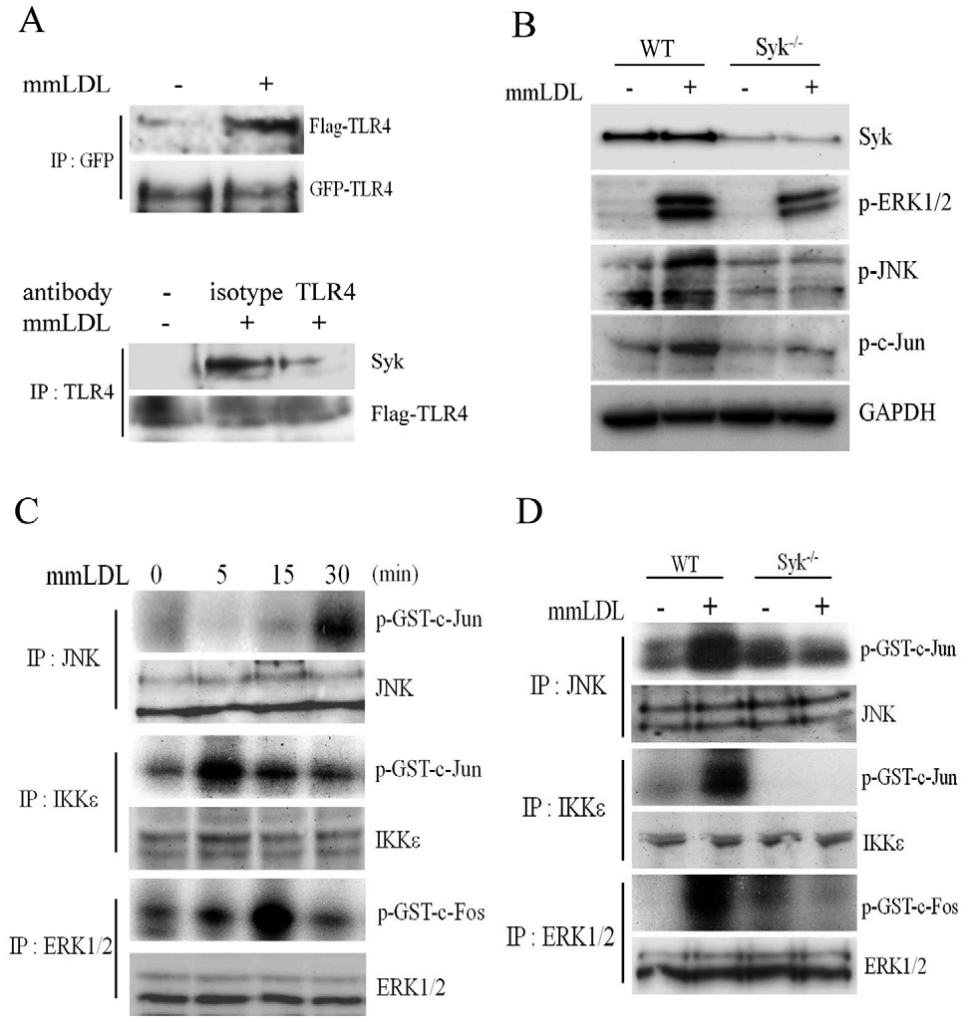
MAP kinases and transcription factors phosphorylation in WT and Syk^−/−^ macrophages stimulated with mmLDL and KLA. (**A**) Upper blots: Ba/F3 cells stably expressing GFP-TLR4 and Flag-TLR4 were incubated with media or mmLDL (50 µg/ml) for 15 min. Cell lysates were immunoprecipitated with an anti-GFP antibody and immunoblotted with anti-Flag and anti-GFP antibodies. Lower blots: Ba/F3 cells were preincubated with 5 µg/ml of an anti-mouse TLR4/MD2 antibody (UT12) or an isotype IgG3κ control for 1 hour and then stimulated with mmLDL (50 µg/ml) for 15 min. Cell lysates were immunoprecipitated with a different anti-mouse TLR4 antibody (UT18) and immunoblotted with anti-Syk and anti-Flag antibodies. (**B**) WT and Syk^−/−^ BMDM were incubated with media or mmLDL (50 µg/ml) for 15 min. Cell lysates were separated on SDS-PAGE and immunoblotted with antibodies against Syk, phospho-ERK1/2, phospho-c-Jun, phospho-JNK and GAPDH. (**C**) *In vitro* kinase assay in J774 macrophages. Cells were incubated with mmLDL (50 µg/ml) for indicated periods of time and then precipitated with anti-JNK, anti-IKKε and anti-ERK1/2 antibodies. Endogenous JNK and IKKε kinase activities were determined using GST-c-Jun (1–79 aa) as a substrate, and endogenous ERK1/2 kinase activity was determined using GST-c-Fos (300–380 aa) as a substrate. Protein levels of endogenous JNK, IKKε and ERK1/2 in the same cell lysates were determined by immunoblot. This experiment informed the optimal stimulation time periods for the experiment in panel D. (**D**) *In vitro* kinase assay in WT and Syk^−/−^ BMDM. Cells were treated with media or mmLDL (50 µg/ml) for 30 min (JNK), 5 min (IKKε) or 15 min (ERK1/2). Kinase activities were determined using the same method as in C.

Syk total knockout mice are perinatally lethal [1]. Thus, we have generated myeloid cell-specific Syk knockout mice by breeding Syk^flox/flox^ mice with LysM-Cre mice. mRNA expression of Syk was undetectable by RT-PCR in BMDM from Syk^−/−^ mice (Syk^flox/flox^ Cre+) compared to WT littermates (Syk^flox/flox^ Cre−) (Fig. 1A). Syk protein levels in both peritoneal macrophages and BMDM were reduced by 60–70% (Fig. 1B; see also Figs. 2B and S1). Because in our earlier studies with Syk shRNA J774 cells we demonstrated that Syk knockdown decreased macrophage lipid accumulation [5], we tested lipid accumulation in Syk^−/−^ BMDM. As expected, lipid accumulation in Syk^−/−^ BMDM was dramatically reduced compared to WT BMDM (Fig. 1C). These results confirm that primary Syk^−/−^ macrophages have a phenotype similar to that observed in a Syk knockdown cell line.

### Syk regulates mmLDL-induced activation of AP-1

An initial step in TLR4 activation is ligand-dependent homodimerization of TLR4. Thus, we tested whether mmLDL induced dimerization of TLR4, using the established model of Ba/F3 cells stably expressing GFP-TLR4 and Flag-TLR4 [13], [14]. As shown in Fig. 2A, in cells incubated with mmLDL, GFP-TLR4 pulled down Flag-TLR4, indicating mmLDL-induced TLR4 dimerization. In addition, TLR4 pulled down Syk as well, reiterating our earlier findings of mmLDL-induced Syk recruitment to TLR4 [4], [5]. Blocking TLR4/MD-2 with a specific antibody diminished mmLDL-induced recruitment of Syk to TLR4 (Fig. 2A).

Next, we used primary Syk-deficient macrophages to evaluate Syk-dependent pathways downstream from TLR4. In BMDM, mmLDL induced phosphorylation of ERK1/2, JNK and c-Jun, and the Syk deficiency dramatically reduced mmLDL-induced phosphorylation of these signaling proteins (Fig. 2B). We also tested Syk^flox/flox^ peritoneal macrophages infected with a Cre adenovirus and confirmed that mmLDL-induced ERK1/2 phosphorylation was Syk-dependent (Fig. S1). mmLDL did not induce phosphorylation of p65 in macrophages (Fig. S2), validating earlier findings that mmLDL does not activate the NF-κB pathway [19].

**Figure 3:**
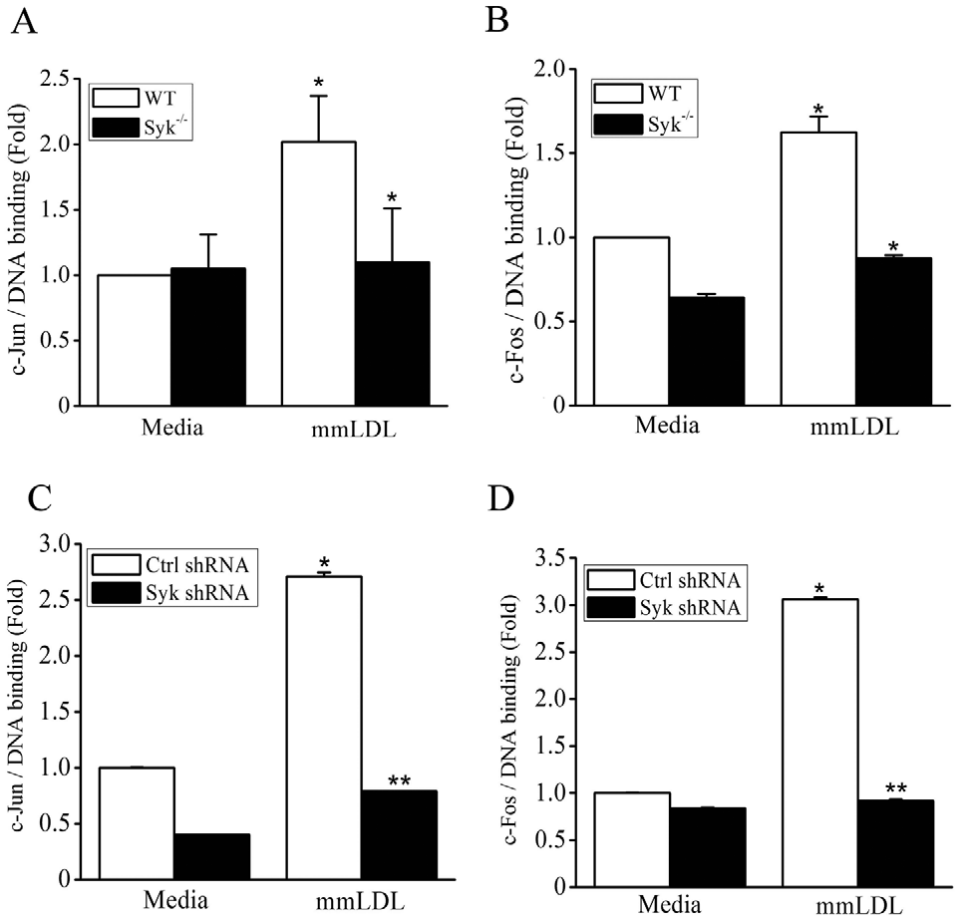
c-Jun and c-Fos DNA binding in WT and Syk^−/−^ macrophages stimulated with mmLDL. BMDM from WT or Syk^−/−^ mice (**A** and **B**) and J774 expressing control of Syk-specific shRNA (**C** and **D**) were incubated for 1 hour with media or 50 µg/ml mmLDL. Nuclear extracts were isolated and used in a transcription factor DNA binding plate-based assay. Mean ± SEM (n = 4). *, p<0.05; **, p<0.005 WT vs. Syk^−/−^.

Phosphorylated c-Jun and c-Fos are major components in the assembly of a heterodimeric AP-1 transcriptional complex. c-Jun is phosphorylated by JNK and IKKε [20]–[22]. Indeed, using an *in vitro* kinase assay, we demonstrated that both JNK and IKKε phosphorylated c-Jun (Figs. 2C and S3). Both phosphorylation events were absolutely dependent on Syk (Fig. 2D). c-Fos is phosphorylated by activated ERK1/2 and p38 [23], [24]. Our previous studies have shown that mmLDL activates ERK1/2, but not p38 [5], [17]. Thus, we performed an *in vitro* kinase assay in lysates precipitated with an ERK1/2 antibody and found that in mmLDL-activated macrophages, ERK1/2 phosphorylated c-Fos and that this process was Syk-dependent as well (Fig. 2C,D). Next, we tested c-Jun and c-Fos binding to consensus DNA sites using a chemiluminescent plate-based assay. For these experiments, we used cell lysates of Syk^−/−^ BMDM and of Syk knockdown J774 cells, with their respective controls. mmLDL induced c-Jun and c-Fos DNA binding in WT cells, and this effect was completely inhibited in Syk-deficient macrophages (Fig. 3).

**Figure 4:**
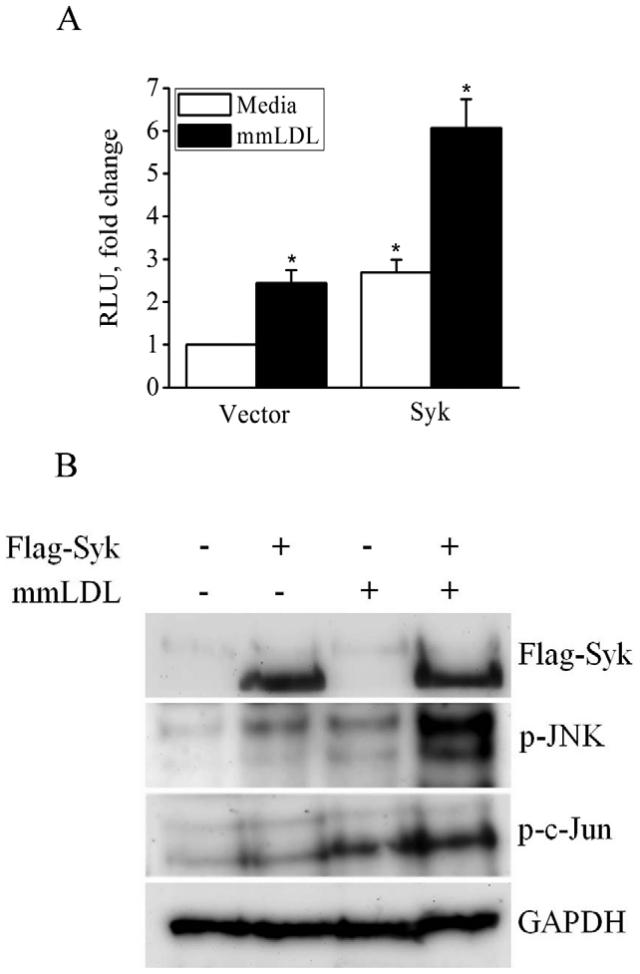
AP-1 activation in Syk-expressing CHO cells. (**A**) CHO cells were transfected with 1 µg Flag-Syk (or empty vector), 200 ng AP-1-Luc reporter, and 200 ng β-galactosidase plasmid. After 36 hours, cells were incubated with media or mmLDL for 6 hours and luciferase activity was measured and normalized to β-galactosidase activity. Mean±SEM (n = 3). *, p<0.05 vector vs. Syk, and media vs. mmLDL. (**B**) CHO Cells were transfected with 300 ng empty vector or Flag-Syk. After 24 hours, cells were incubated with mmLDL for 6 hours and cell lysates were tested for expression of Syk (Flag) and for p-JNK, p-c-Jun and GAPDH.

In gain-of-function experiments, CHO cells were transiently transfected with a Syk expression plasmid (or empty vector) and a AP-1-Luc reporter. Because CHO cells express endogenous TLR4/MD2 [25], mmLDL induced a 2-fold increase in AP-1 activity in empty vector-transfected cells. Overexpression of Syk potentiated mmLDL-induced AP-1 activity and increased it additional 3-fold (Fig. 4A). Furthermore, Syk expression induced phosphorylation of JNK and c-Jun in mmLDL-stimulated CHO cells (Fig. 4B). These results collectively suggest that Syk regulates a AP-1 transcription program in macrophages.

### Syk regulates expression of CXCL2 and IL-6 induced by mmLDL

**Figure 5:**
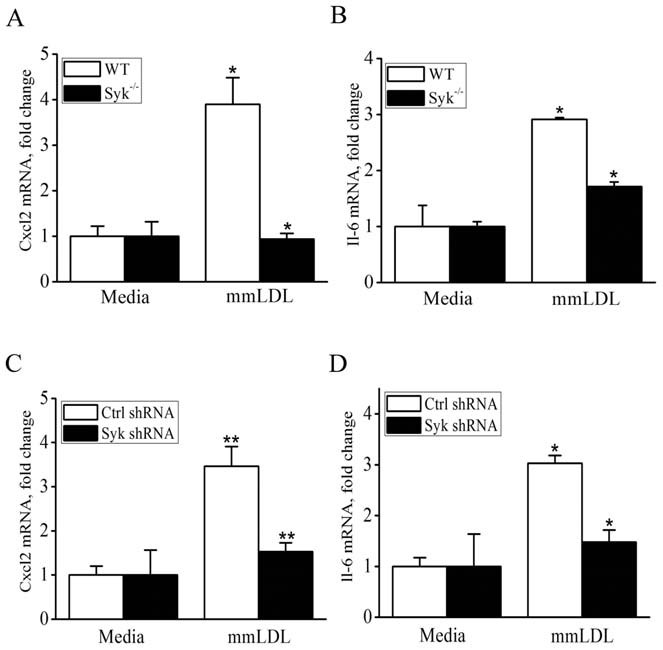
mRNA expression of Cxcl2 and Il-6 in WT and Syk^−/−^ macrophages stimulated with mmLDL. BMDM from WT or Syk^−/−^ mice (**A** and **B**) and J774 cells expressing control or Syk-specific shRNA (**C** and **D**) were incubated for 1 hour with media or 50 µg/ml mmLDL. Total RNA was isolated and expression of Cxcl2 and Il-6 mRNA was measured by qPCR and normalized to the GAPDH mRNA levels. Mean ± SEM (n = 3). *, p<0.05; **, p<0.005 WT vs. Syk^−/−^.

Among AP-1 dependent genes, our previous studies identified *Cxcl2* (MIP-2) as one of the best responders to mmLDL activation in macrophages [4], [5], [26], [27]. Transcription of IL-6 is also AP-1-dependent, and it has a strong NF-κB component as well [28]. In experiments shown in Fig. 5, mmLDL induced Cxcl2 and Il-6 mRNA expression both in WT BMDM and in WT J774 cells. The Syk deficiency in BMDM completely abolished mmLDL-induced Cxcl2 expression and reduced in half expression of Il-6.

**Figure 6:**
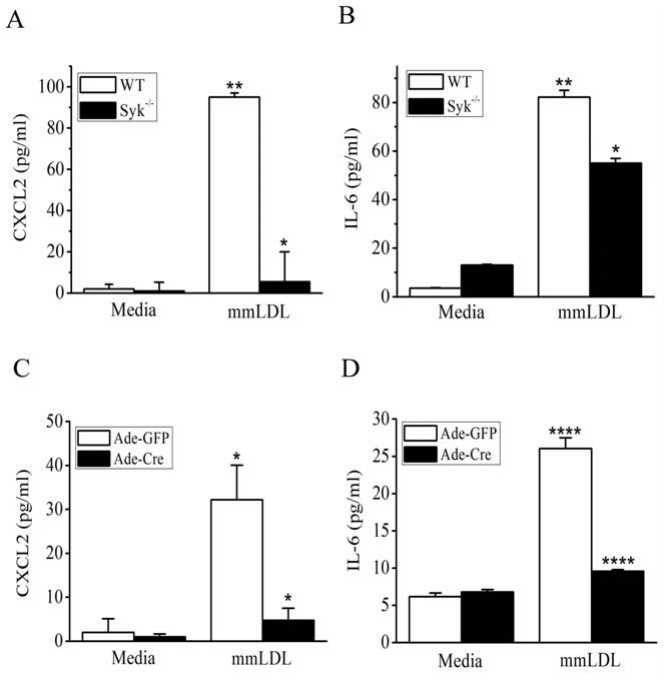
Secretion of CXCL2 (MIP-2) and IL-6 by WT and Syk^−/−^ macrophages stimulated with mmLDL. BMDM from WT or Syk^−/−^ mice (**A** and **B**) and resident peritoneal macrophages from Syk^flox/flox^ mice infected with adenovirus expressing GFP or Cre (for 48 hours at 500 MOI) (**C** and **D**) were incubated for 6 hours with media or 50 µg/ml mmLDL. Cell culture media were collected and CXCL2 and IL-6 protein levels were measured by ELISA. Mean ± SEM (n = 3). *, p<0.05; ****, p<0.00005 WT vs. Syk^−/−^.

Next, for testing the effect of Syk deficiency on secretion of CXCL2 (MIP-2) and IL6 protein, we used two types of primary macrophages: Syk^−/−^ BMDM and peritoneal macrophages from Syk^flox/flox^ mice infected *in vitro* with a Cre adenovirus (or with a GFP adenovirus as a control). In agreement with the mRNA levels (Fig. 5), mmLDL-induced secretion of CXCL2 was absolutely Syk-dependent, while secretion of IL-6 was reduced by 40–80% in Syk-deficient macrophages (Fig. 6). Graphs in Fig. 6 show the cytokine levels 6 hours after mmLDL stimulation, and graphs in Fig. S4 show cytokine levels after 24 hours.

## Discussion

Using mmLDL as an activator of TLR4-dependent responses in primary macrophages, in this study we demonstrated that Syk regulates ERK1/2-dependent phosphorylation of c-Fos and JNK- and IKKε-dependent phosphorylation of c-Jun, leading to AP-1-dependent expression of proinflammatory cytokines. Although involvement of Syk in macrophage responses to LPS have been reported [29], [30], uncovering the mechanism of Syk in TLR4-dependent transcriptional regulation became possible due to the fact that mmLDL, unlike LPS, does not induce any apparent MyD88-mediated responses [4], [5]. In this study, we also observed MyD88-independent phosphorylation of ERK1/2 induced by mmLDL in macrophages (Fig. S5). The mechanism explaining lack of MyD88 activation by mmLDL is yet to be found. However, the use of mmLDL presents a tool for dissecting MyD88-independent pathways in experiments in which absence of a strong MyD88/NF-κB component does not mask other pathways downstream from TLR4.

As we suggested in a recent review article [8], pattern recognition receptors (PRRs), such as TLR4, likely evolved in invertebrates and early vertebrates to control homeostasis and development. Studies from our group and the studies of others have demonstrated that oxidation-specific epitopes formed in apoptotic cells and in oxidized lipoproteins (mmLDL is an example) are recognized by PRRs of innate immunity (summarized in [8]). Responses to these host-derived damage-associated molecular patterns (DAMPs) involve homeostatic surveillance of the tissues, characterized by extensive cytoskeletal changes and macropinocytosis/phagocytosis and moderate secretion of inflammatory cytokines. Our results showing mmLDL activation of the TLR4/Syk signaling pathway, resulting in macropinocytosis [5] and moderate AP-1 dependent secretion of CXCL2 and IL-6 (this study) support this hypothesis.

Because many of the same oxidation-specific PRRs also recognize pathogen-associated molecular patterns (PAMPs), we speculated that the ability to protect against infectious pathogens provided a strong secondary selecting pressure for PRRs. For the purpose of host defense against microbial pathogens, stronger cellular responses were required to such infectious pathogens than those resulting from host-derived activation. Indeed, LPS-induced TLR4 activation results in an extremely strong MyD88-dependent NF-κB transcriptional response. This implies that the intracellular signaling pathways linking a PRR response to an endogenous DAMP may be different from those involved in the same PRR response to microbial PAMPs. This paper provides such an example showing MyD88-independent signaling via Syk in mmLDL-activated macrophages, resulting in a relatively muted cytokine response. However, chronic exposure to increased levels of oxidation-specific DAMPs, such as mmLDL in hypercholesterolemic subjects, likely induces low-grade but sustained activation of PRRs, e.g. TLR4 and its Syk signaling component, and thus, may contribute to the inflammatory state in atherosclerotic lesions.

Understanding cellular mechanisms of Syk regulation has the immediate importance because the oral Syk inhibitor fostamatinib is currently in a phase III clinical trial in patients with rheumatoid arthritis. Several companies develop a number of new Syk inhibitors for various clinical applications [1]. Although targeting Syk is currently designed to benefit conditions with excessively upregulated ITAM-mediated responses, such as rheumatoid arthritis, allergies, asthma, lupus, etc., our findings and other reports point to the importance of Syk in regulation of TLR4 responses, particularly by host-derived ligands, and development of chronic inflammatory diseases, such as atherosclerosis. Indeed, a very recent report by Hilgendorf et al. [31] demonstrates that administering fostamatinib attenuates atherosclerosis in Ldlr−/− mice fed a high-cholesterol diet. The results of the current study as well as macrophage-specific Syk^−/−^ mice generated in our laboratory will be useful in addressing mechanisms involved in Syk-dependent chronic inflammation and atherosclerosis.

## Supporting Information

Figure S1 **ERK1/2 phosphorylation in Adeno-Cre/Syk^flox/flox^ macrophages stimulated with mmLDL.** Resident peritoneal macrophages from Syk^flox/flox^ mice were infected with adenovirus expressing either GFP or Cre for 48 hours at 500 MOI and then incubated with media or 50 µg/ml mmLDL for 15, 30 or 60 min. Cell lysates were separated on Nu-PAGE and immunoblotted with antibodies against Syk, phospho-ERK1/2 or GAPDH.
(TIF)

Figure S2 **Lack of p65 phopshorylation in response to mmLDL.** WT and Syk^−/−^ BMDM were incubated with media or mmLDL (50 µg/ml) for 15 min. Cell lysates were separated on SDS-PAGE and immunoblotted with antibodies against phospho-p65 and GAPDH.
(TIF)

Figure S3 **Role of JNK, IKKε and ERK1/2 in phosphorylation of c-Jun and c-Fos in mmLDL-stimulated macrophages: Quantification of results presented in** **Fig. 2C** **.** *In vitro* kinase assay in J774 macrophages. Cells were incubated with mmLDL (50 µg/ml) for indicated periods of time and then precipitated with anti-JNK, anti-IKKε and anti-ERK1/2 antibodies. Endogenous JNK and IKKε kinase activities were determined using GST-c-Jun (1–79 aa) as a substrate, and endogenous ERK1/2 kinase activity was determined using GST-c-Fos (300–380 aa) as a substrate. Band densities for p-c-Jun and p-c-Fos were normalized to JNK, IKKε and ERK1/2 in corresponding blots. Mean±SD from two independent experiments.
(TIF)

Figure S4 **Secretion of CXCL2 (MIP-2) and IL-6 by WT and Syk^−/−^ macrophages stimulated with mmLDL.** BMDM from WT or Syk^−/−^ mice (**A** and **B**) were incubated for 24 hours with media or 50 µg/ml mmLDL. Cell culture media were collected and CXCL2 and IL-6 protein levels were measured by ELISA. Mean ± SEM from 3 independent experiments. *, p<0.05; ***, p<0.0005 WT vs. Syk^−/−^.
(TIF)

Figure S5 **mmLDL-induced phosphorylation of ERK1/2 in WT and MyD88^−/−^ macrophages.** WT and MyD88^−/−^ BMDM (mouse genotypes have been confirmed with PCR) were incubated with media or mmLDL (50 µg/ml) for 15 min. Cell lysates were separated on SDS-PAGE and immunoblotted with antibodies against phospho-ERK1/2 and GAPDH.
(TIF)

## References

[1] Mocsai A, Ruland J, Tybulewicz VLJ (2010). The SYK tyrosine kinase: a crucial player in diverse biological functions.. Nat Rev Immunol.

[2] Jakus Z, Fodor S, Abram CL, Lowell CA, Mocsai A (2007). Immunoreceptor-like signaling by [beta]2 and [beta]3 integrins.. Trends in Cell Biology.

[3] Woodside DG, Obergfell A, Talapatra A, Calderwood DA, Shattil SJ (2002). The N-terminal SH2 Domains of Syk and ZAP-70 Mediate Phosphotyrosine-independent Binding to Integrin Cytoplasmic Domains.. J Biol Chem.

[4] Bae YS, Lee JH, Choi SH, Kim S, Almazan F (2009). Macrophages Generate Reactive Oxygen Species in Response to Minimally Oxidized Low-Density Lipoprotein: Toll-Like Receptor 4- and Spleen Tyrosine Kinase-Dependent Activation of NADPH Oxidase 2.. Circ Res.

[5] Choi S-H, Harkewicz R, Lee JH, Boullier A, Almazan F (2009). Lipoprotein accumulation in macrophages via toll-like receptor-4-dependent fluid phase uptake.. Circ Res.

[6] Miller YI, Choi SH, Fang L, Harkewicz R (2009). Toll-like receptor-4 and lipoprotein accumulation in macrophages.. Trends Cardiovasc Med.

[7] Stoletov K, Fang L, Choi SH, Hartvigsen K, Hansen LF (2009). Vascular Lipid Accumulation, Lipoprotein Oxidation, and Macrophage Lipid Uptake in Hypercholesterolemic Zebrafish.. Circ Res.

[8] Miller YI, Choi S-H, Wiesner P, Fang L, Harkewicz R (2011). Oxidation-Specific Epitopes are Danger Associated Molecular Patterns Recognized by Pattern Recognition Receptors of Innate Immunity.. Circ Res.

[9] Saijo K, Schmedt C, Su Ih, Karasuyama H, Lowell CA (2003). Essential role of Src-family protein tyrosine kinases in NF-[kappa]B activation during B cell development.. Nat Immunol.

[10] Kawai T, Adachi O, Ogawa T, Takeda K, Akira S (1999). Unresponsiveness of MyD88-deficient mice to endotoxin.. Immunity.

[11] Sawka-Verhelle D, Escoubet-Lozach L, Fong AL, Hester KD, Herzig S (2004). PE-1/METS, an Antiproliferative Ets Repressor Factor, Is Induced by CREB-1/CREM-1 during Macrophage Differentiation.. J Biol Chem.

[12] Benz DJ, Mol M, Ezaki M, Mori-Ito N, Zelaan I (1995). Enhanced levels of lipoperoxides in low density lipoprotein incubated with murine fibroblast expressing high levels of human 15-lipoxygenase.. J Biol Chem.

[13] Saitoh S, Akashi S, Yamada T, Tanimura N, Kobayashi M (2004). Lipid A antagonist, lipid IVa, is distinct from lipid A in interaction with Toll-like receptor 4 (TLR4)-MD-2 and ligand-induced TLR4 oligomerization.. International Immunology.

[14] Wong SW, Kwon MJ, Choi AMK, Kim HP, Nakahira K (2009). Fatty Acids Modulate Toll-like Receptor 4 Activation through Regulation of Receptor Dimerization and Recruitment into Lipid Rafts in a Reactive Oxygen Species-dependent Manner.. J Biol Chem.

[15] Havel RJ, Bragdon JH, Eder HA (1955). The distribution and chemical composition of ultracentrifugally separated lipoproteins in human serum.. J Clin Invest.

[16] Miller YI, Viriyakosol S, Binder CJ, Feramisco JR, Kirkland TN (2003). Minimally Modified LDL Binds to CD14, Induces Macrophage Spreading via TLR4/MD-2, and Inhibits Phagocytosis of Apoptotic Cells.. J Biol Chem.

[17] Miller YI, Viriyakosol S, Worrall DS, Boullier A, Butler S (2005). Toll-like receptor 4-dependent and -independent cytokine secretion induced by minimally oxidized low-density lipoprotein in macrophages.. Arterioscler Thromb Vasc Biol.

[18] Boullier A, Li Y, Quehenberger O, Palinski W, Tabas I (2006). Minimally oxidized LDL offsets the apoptotic effects of extensively oxidized LDL and free cholesterol in macrophages.. Arterioscler Thromb Vasc Biol.

[19] Wiesner P, Choi SH, Almazan F, Benner C, Huang W (2010). Low Doses of Lipopolysaccharide and Minimally Oxidized Low-Density Lipoprotein Cooperatively Activate Macrophages via Nuclear Factor {kappa}B and Activator Protein-1. Possible Mechanism for Acceleration of Atherosclerosis by Subclinical Endotoxemia.. Circ Res.

[20] Davis RJ (2000). Signal Transduction by the JNK Group of MAP Kinases.. Cell.

[21] Huang W, Ghisletti S, Perissi V, Rosenfeld MG, Glass CK (2009). Transcriptional Integration of TLR2 and TLR4 Signaling at the NCoR Derepression Checkpoint.. Molecular Cell.

[22] Sweeney SE, Hammaker D, Boyle DL, Firestein GS (2005). Regulation of c-Jun Phosphorylation by the IkappaB Kinase-epsilon Complex in Fibroblast-Like Synoviocytes.. The Journal of Immunology.

[23] Murphy LO, Smith S, Chen RH, Fingar DC, Blenis J (2002). Molecular interpretation of ERK signal duration by immediate early gene products.. Nat Cell Biol.

[24] Tanos T, Marinissen MJ, Leskow FC, Hochbaum D, Martinetto H (2005). Phosphorylation of c-Fos by Members of the p38 MAPK Family.. J Biol Chem.

[25] Schromm AB, Lien E, Henneke P, Chow JC, Yoshimura A (2001). Molecular Genetic Analysis of an Endotoxin Nonresponder Mutant Cell Line.. J Exp Med.

[26] Miller YI (2005). Toll-like receptors and atherosclerosis: oxidized LDL as an endogenous Toll-like receptor ligand.. Future Cardiology.

[27] Harkewicz R, Hartvigsen K, Almazan F, Dennis EA, Witztum JL (2008). Cholesteryl ester hydroperoxides are biologically active components of minimally oxidized LDL.. J Biol Chem.

[28] Faggioli L, Costanzo C, Donadelli M, Palmieri M (2004). Activation of the interleukin-6 promoter by a dominant negative mutant of c-Jun.. Biochimica et Biophysica Acta (BBA) - Molecular Cell Research.

[29] Ulanova M, Asfaha S, Stenton G, Lint A, Gilbertson D (2007). Involvement of Syk protein tyrosine kinase in LPS-induced responses in macrophages.. J Endotoxin Res.

[30] Lin YC, Huang DY, Chu CL, Lin WW (2010). Anti-inflammatory actions of Syk inhibitors in macrophages involve non-specific inhibition of toll-like receptors-mediated JNK signaling pathway.. Mol Immunol.

[31] Hilgendorf I, Eisele S, Remer I, Schmitz J, Zeschky K (2011). The Oral Spleen Tyrosine Kinase Inhibitor Fostamatinib Attenuates Inflammation and Atherogenesis in Low-Density Lipoprotein Receptor-Deficient Mice.. Arterioscler Thromb Vasc Biol.

