# Supplementary figures and images for: Spleen Tyrosine Kinase Regulates AP-1 Dependent Transcriptional Response to Minimally Oxidized LDL
Source: PLoS One. 2012 Feb 22;7(2):e32378. doi: 10.1371/journal.pone.0032378 (PMC3284564; doi:10.1371/journal.pone.0032378)

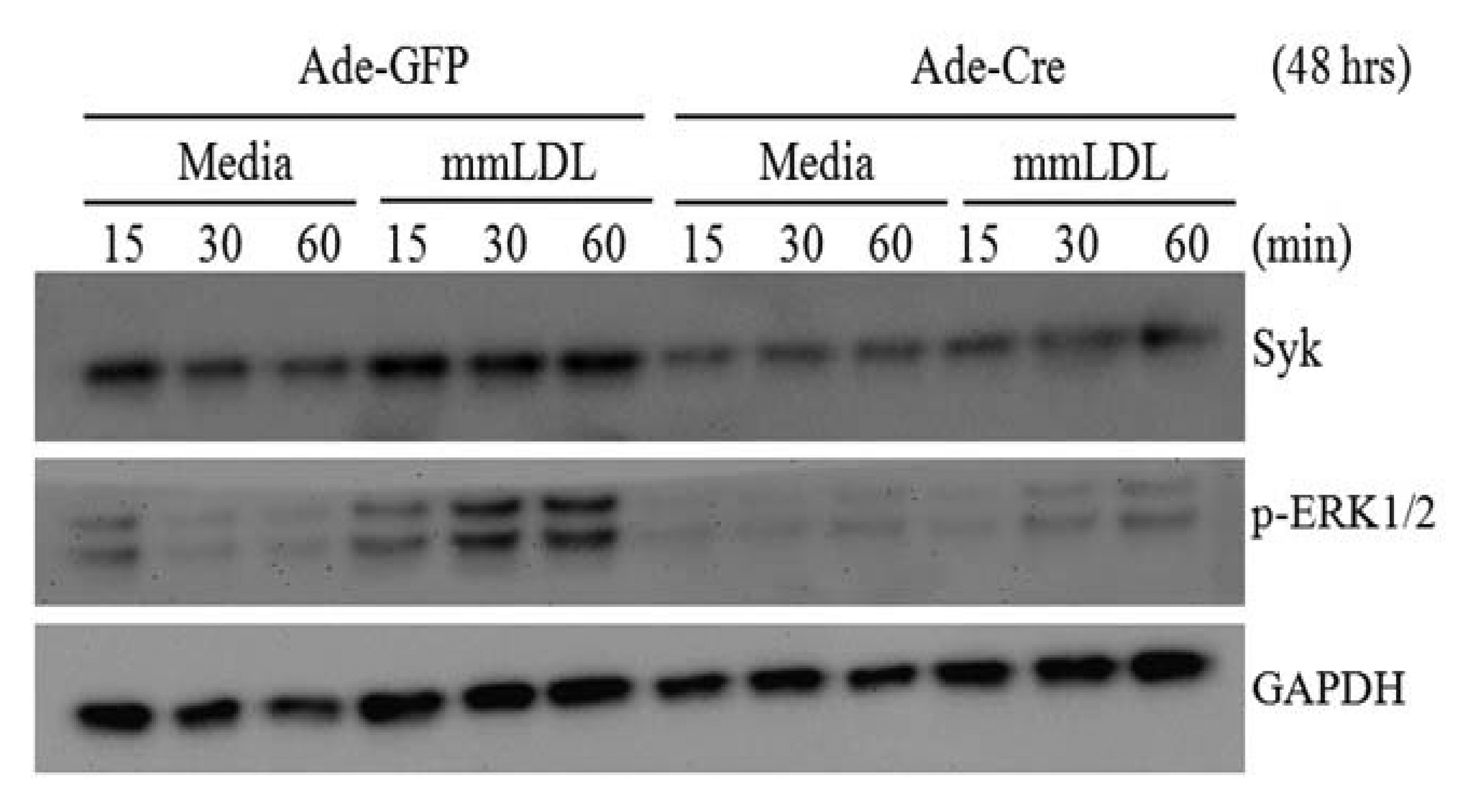

Supplement: Figure S1 — ERK1/2 phosphorylation in Adeno-Cre/Sykflox/flox macrophages stimulated with mmLDL. Resident peritoneal macrophages from Sykflox/flox mice were infected with adenovirus expressing either GFP or Cre for 48 hours at 500 MOI and then incubated with media or 50 µg/ml mmLDL for 15, 30 or 60 min. Cell lysates were separated on Nu-PAGE and immunoblotted with antibodies against Syk, phospho-ERK1/2 or GAPDH. (TIF) [file pone.0032378.s001.tif]

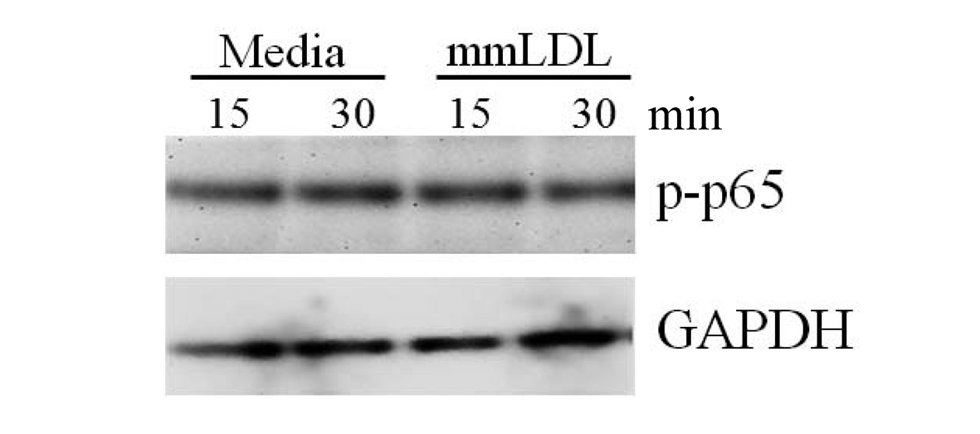

Supplement: Figure S2 — Lack of p65 phopshorylation in response to mmLDL. WT and Syk−/− BMDM were incubated with media or mmLDL (50 µg/ml) for 15 min. Cell lysates were separated on SDS-PAGE and immunoblotted with antibodies against phospho-p65 and GAPDH. (TIF) [file pone.0032378.s002.tif]

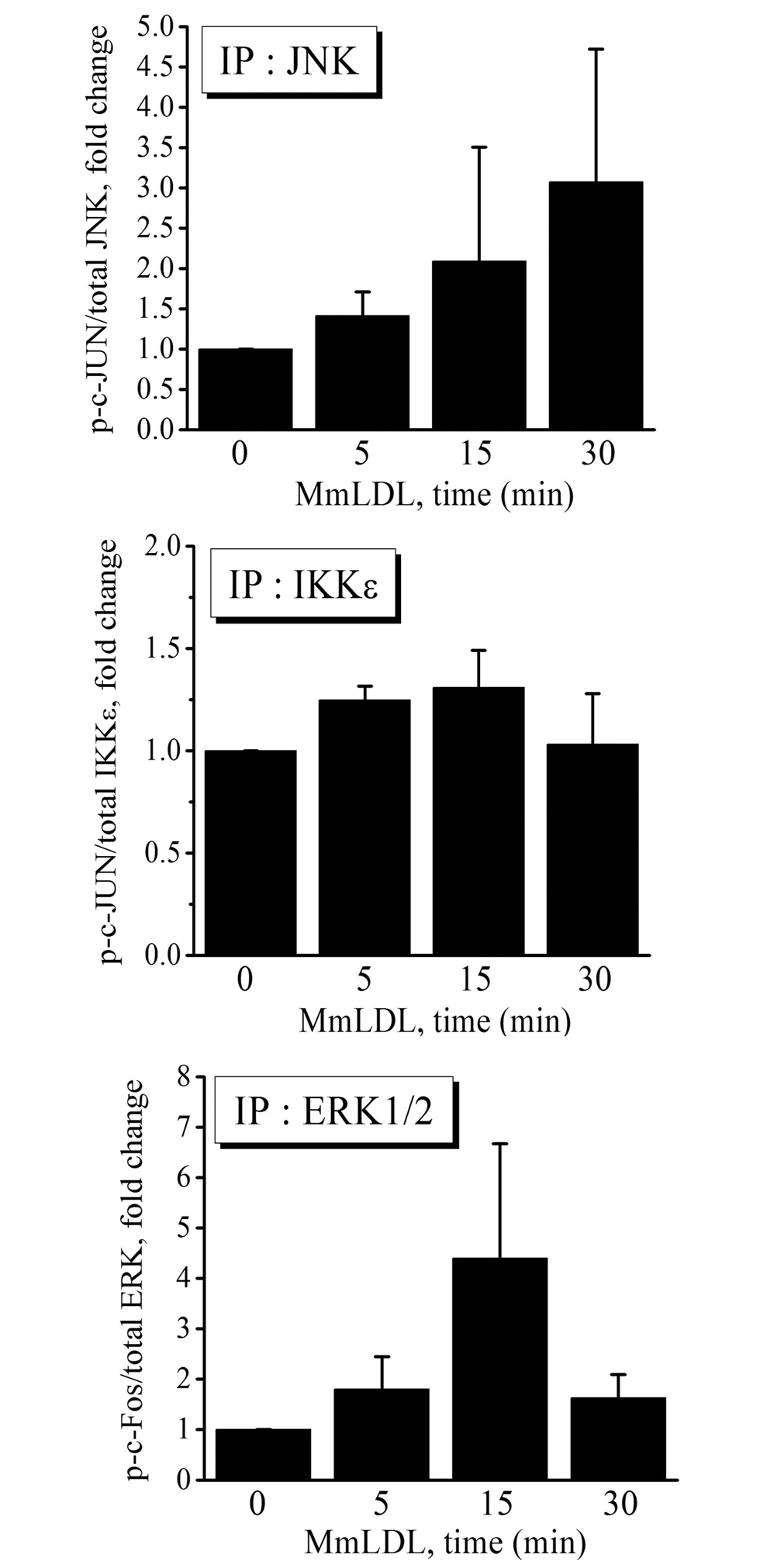

Supplement: Figure S3 — Role of JNK, IKKε and ERK1/2 in phosphorylation of c-Jun and c-Fos in mmLDL-stimulated macrophages: Quantification of results presented in Fig. 2C . In vitro kinase assay in J774 macrophages. Cells were incubated with mmLDL (50 µg/ml) for indicated periods of time and then precipitated with anti-JNK, anti-IKKε and anti-ERK1/2 antibodies. Endogenous JNK and IKKε kinase activities were determined using GST-c-Jun (1–79 aa) as a substrate, and endogenous ERK1/2 kinase activity was determined using GST-c-Fos (300–380 aa) as a substrate. Band densities for p-c-Jun and p-c-Fos were normalized to JNK, IKKε and ERK1/2 in corresponding blots. Mean±SD from two independent experiments. (TIF) [file pone.0032378.s003.tif]

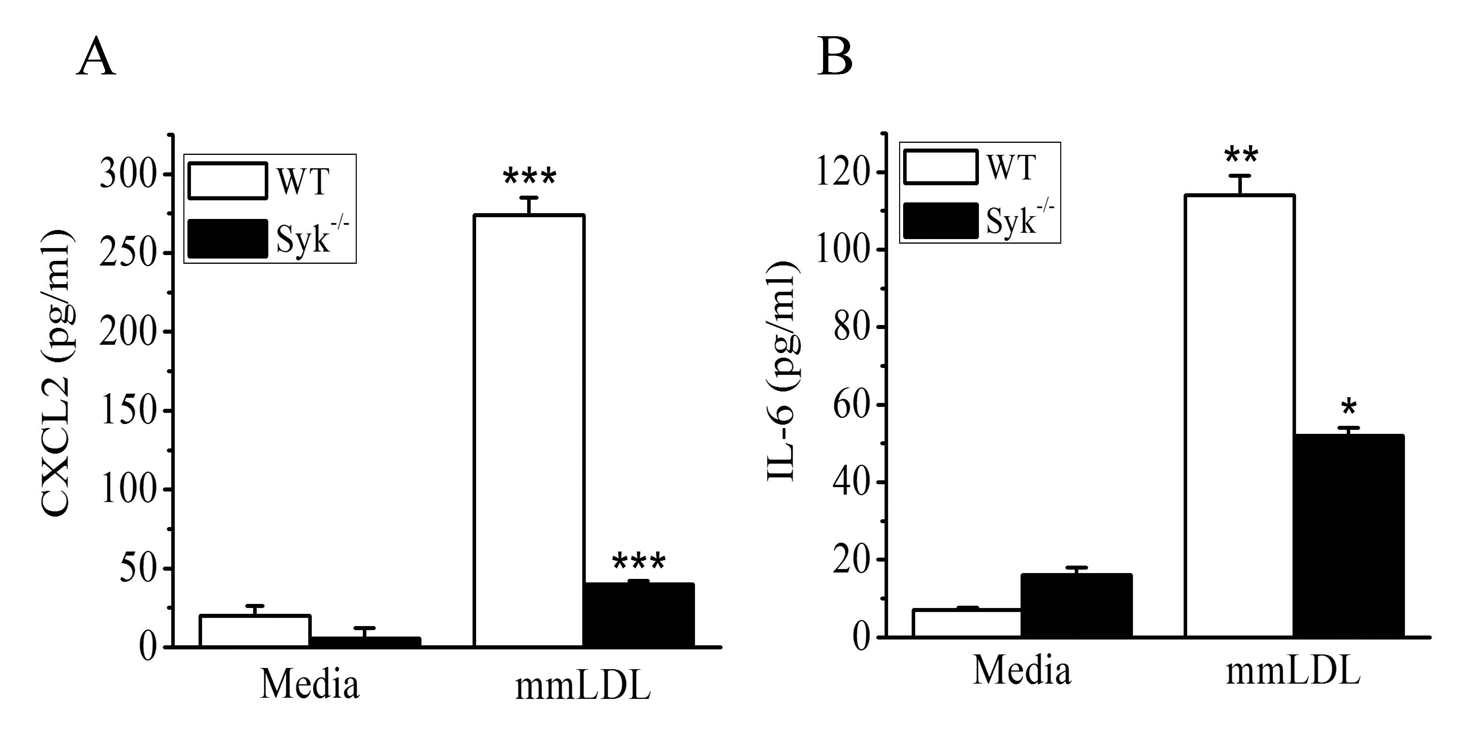

Supplement: Figure S4 — Secretion of CXCL2 (MIP-2) and IL-6 by WT and Syk−/− macrophages stimulated with mmLDL. BMDM from WT or Syk−/− mice (A and B) were incubated for 24 hours with media or 50 µg/ml mmLDL. Cell culture media were collected and CXCL2 and IL-6 protein levels were measured by ELISA. Mean ± SEM from 3 independent experiments. *, p<0.05; ***, p<0.0005 WT vs. Syk−/−. (TIF) [file pone.0032378.s004.tif]

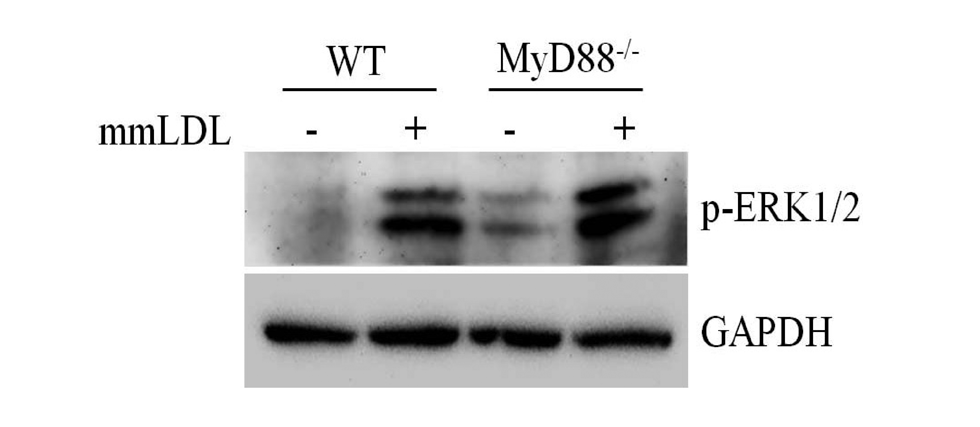

Supplement: Figure S5 — mmLDL-induced phosphorylation of ERK1/2 in WT and MyD88−/− macrophages. WT and MyD88−/− BMDM (mouse genotypes have been confirmed with PCR) were incubated with media or mmLDL (50 µg/ml) for 15 min. Cell lysates were separated on SDS-PAGE and immunoblotted with antibodies against phospho-ERK1/2 and GAPDH. (TIF) [file pone.0032378.s005.tif]
